# Free Spectacles for Children in Public Schools

**Published:** 1906-07-28

**Authors:** 


### EDITOR'S LETTER-BOX.

[Our Correspondents are reminded that prolixity is a great bar to publication, and that brevity of style and conciseness of statement greatly facilitate early insertion.]

### FREE SPECTACLES FOR CHILDREN IN PUBLIC SCHOOLS.

"A medical officer of schools" writes: Your editorial note upon the letter of Miss Susan Lawrence, which dealt with the subject of supplying spectacles to children seems to contain two contradictory conclusions. The note says that it is the "plain duty" of the Legislature to provide "necessaries" to children "when they are not supplied by parents"; but of the Association for the supply of one of these necessaries it says, "the whole business" is a device directed towards "the more complete demoralisation" c the lower classes. One cannot but think that if it is demoralising for an association to supply spectacles it is demoralising also for the Legislature to provide them, and I can hardly suppose that you consider it to be the "plain duty" of the Legislature to aid in demoralisation. But, leaving this enigma, it is evident that you admit that there are children who need spectacles who do not get them. Whether you also admit that there are children who have parents that cannot afford them is not so clear; but it may be pointed out that many children are dependent upon a widowed mother, or, perhaps, not even upon her, they may have been taken into the already large family of a brother, sister, or friend of the deceased mother or father. You speak, however, of the selection of cases by managers, but consider that it would "inaugurate a reign of chaos." In this connection it may be remarked that the managers would no doubt consult the school-teachers, and there is no body of men and women in England who know so much of the circumstances of the home life of children attending schools.

. ˙ . The "contradiction" is only in the mind of our correspondent. If spectacles are "necessaries" it is the business of the State to supply them in any case in which the inability of the parent is established by proper tests, which should be uniform and applied under official supervision. If private charity is to supply "necessaries," they will be supplied in some cases or in some localities, and withheld in others.—Ed. *T. H.*

